# Prediction of the Future Evolution Trends of *Prunus sibirica* in China Based on the Key Climate Factors Using MaxEnt Modeling

**DOI:** 10.3390/biology13120973

**Published:** 2024-11-25

**Authors:** Jiazhi Wang, Jiming Cheng, Chao Zhang, Yingqun Feng, Lang Jin, Shuhua Wei, Hui Yang, Ziyu Cao, Jiuhui Peng, Yonghong Luo

**Affiliations:** 1 China Meteorological Administration Xiong’an Atmospheric Boundary Layer Key Laboratory, Xiong’an New Area 071800, China; george0602@126.com; 2 Key Laboratory of Meteorology and Ecological Environment of Hebei Province, Shijiazhuang 050021, China; 3 Chengde Meteorological Bureau, Chengde 067000, China; 4 School of Life Sciences, Central China Normal University, Wuhan 430079, China; chengjiming@mails.ccnu.edu.cn (J.C.); zhangchao2023@mails.ccnu.edu.cn (C.Z.); fyq0016094@163.com (Y.F.); jinlang2023@mails.ccnu.edu.cn (L.J.); 5 College of Biological Science and Engineering, North Minzu University, Yinchuan 750021, China; yh20217525@163.com (H.Y.); czy200218@163.com (Z.C.); 6 Ningxia Academy of Agriculture and Forestry Sciences, Plant Protection Institute, Yinchuan 750021, China; weishuhua666@163.com

**Keywords:** climate change, MaxEnt, precipitation, *Prunus sibirica*, species distribution modeling

## Abstract

Mountain apricot (*Prunus sibirica*) is an important fruit tree variety, and has a wide range of planting and application value in China and even the world. However, the current research on the suitable distribution area of *P. sibirica* is still inconclusive. In this study, we retrieved distribution data for *P. sibirica* in China from the Global Biodiversity Information Facility (GBIF), and identified six key environmental factors influencing its distribution through cluster analysis. Using these six selected climate factors and *P. sibirica* distribution points in China, we applied the maximum entropy model (MaxEnt) to evaluate 1160 candidate models for parameter optimization. The final results predict the potential distribution of *P. sibirica* under the current climate as well as two future climate scenarios (SSPs126 and SSPs585). This study shows that the model optimized with six key climate factors (AUC = 0.897, TSS = 0.658) outperforms the full model using nineteen climate factors (AUC = 0.894, TSS = 0.592). Under the high-emission scenario (SSPs585), the highly suitable habitat for *P. sibirica* is expected to gradually shrink towards the southeast and northwest, while expanding in the northeast and southwest. After the 2050s, highly suitable habitats are projected to completely disappear in Shandong, while new suitable areas may emerge in Tibet. Additionally, the total area of suitable habitat is projected to increase in the future, with a more significant expansion under the high-emission scenario (SSPs585) compared to the low-emission scenario (SSPs126) (7.33% vs. 0.16%). Seasonal changes in precipitation are identified as the most influential factor in driving the distribution of *P. sibirica*.

## Simple Summary

We used the MaxEnt model to predict the suitable distribution area of mountain apricot (*Prunus sibirica*). We found that climate change will significantly impact the distribution of wild apricots, particularly under high-emission scenarios, where their suitable habitats are expected to shift considerably. These findings provide valuable insights into the conservation and management of wild apricots, while also contributing to a broader understanding of how climate change influences plant distributions.

## 1. Introduction

Global climate change has attracted the attention of many ecologists and biologists. Climate change has become one of the most urgent ecological and environmental problems in the world. Climate change not only affects temperature and precipitation patterns, but also leads to a variety of ecological problems, such as an increase in extreme weather events, rising sea levels, and reduced biodiversity [1,2]. As plants are fundamental components of the ecosystem, changes in their distribution range and growth status will directly affect the function and stability of the whole ecosystem [3,4]. However, climate change leads to changes in various environmental factors, which seriously affect the distribution and growth of plants [5,6,7]. As the global climate continues to shift, it becomes increasingly important to understand how these environmental changes influence plant species and their potential for adaptation.

The maximum entropy model (MaxEnt) is often used to analyze and predict species distribution [8,9]. The MaxEnt model has higher modeling accuracy than other models, and achieves better predictions of existing areas, determining latitude and longitude in the absence of clear species distribution coordinates [6,10,11]. Therefore, the MaxEnt model is one of the most effective prediction methods for species distributions [1,12,13]. However, the MaxEnt model has some limitations, such as excessive environmental factors that may cause redundancy in the model [7,14,15,16,17]. Thus, it is particularly important to optimize the MaxEnt model [18,19]. For example, the best climate predictors can be selected to further improve the accuracy and reliability of the model. Moreover, the input parameters of the model are also important factors affecting the predictive ability of the model. Cobos et al. (2019) proposed an optimization method for model parameters, which was proven to simplify the MaxEnt model and significantly improve the predictive ability of the model [20]. We can use the optimized model to compare with the original model, leading to more accurate and reliable conclusions [7,20]. By applying this optimized model, we can make more accurate predictions for plant species, particularly those that thrive under harsh climatic conditions and hold significant ecological and economic value. 

Mountain apricot (*Prunus sibirica*) is an important fruit tree variety with wide-ranging planting and application value in China and globally. Due to its ecological benefits, such as water and soil conservation, windbreak ability, sand fixation, and environmental protection, it is commonly used for afforestation efforts in northern China [21,22,23]. Mountain apricot not only holds significant economic and ecological value but is also widely used in traditional Chinese medicine. Its seed kernels are employed in various preparations to treat ailments such as asthma, coughs, and viral pneumonia in infants [24,25]. However, with climate change, the habitat environment of the mountain apricot is also changing [26]. The environment in which apricot grows and survives is influenced not only by atmospheric temperature and precipitation, but also by various factors such as soil temperature, water and pH [27,28]. To better understand the response to climate change, we selected the maximum entropy model (MaxEnt) and predicted the distribution in future climate conditions by optimizing the MaxEnt model and screening environmental factors [7,20]. We hypothesize that a small set of key climatic factors can effectively predict the current and future potential distribution of *Prunus sibirica* using the MaxEnt model. From an ecological perspective, these factors will help explain why wild apricot is able to survive and reproduce in harsh environments, and ultimately become a dominant species. This study provides a deeper understanding and effective coping strategies for biodiversity conservation and ecosystem management.

## 2. Materials and Methods

### 2.1. Study Area

The study areas are situated across regions in China with diverse climates and topography, including tropical, subtropical, temperate, and Qinghai–Tibetan Plateau zones. The southern lowlands are characterized by warm, humid climates that support tropical and subtropical forests, while the central and northern mountainous regions have temperate climates, with temperate forests and alpine vegetation. The high-altitude Qinghai–Tibetan Plateau presents harsh conditions, fostering unique alpine ecosystems. These variations in climate and topography provide an ideal setting for studying the impacts of climate change, particularly for species like wild apricot, which thrive in extreme environments. This supports essential functions such as water and soil conservation [29], desertification control [30], ecological restoration and reconstruction [31], and contributes to multiple ecosystem services [32], highlighting its ecological significance.

### 2.2. Species Occurrence Data

**Figure 1:**
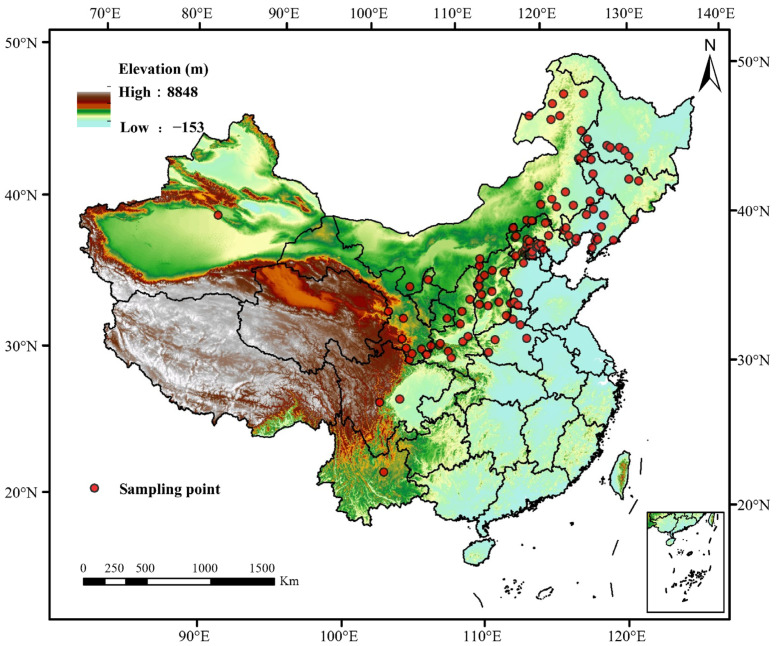
Geographical map of the distribution points of *Prunus sibirica*.

Mountain apricot distribution point data in China were retrieved using the global bioinformation platform (GBIF, https://www.gbif.org/), and 309 records were collected. The data were then collated regarding species names, longitude and latitude. To make the prediction results more accurate, we removed redundant data and duplicate data from the data using the ENMTools (https://github.com/danlwarren/ENMTools, last accessed on 27 March 2024) program. Finally, the effective distribution point data of 132 items of mountain apricots were obtained (Figure 1).

### 2.3. Environmental Variables

This study used modern climate data (1970–2000) and future climate data, which were obtained from the World Climate Data website (WorldClim, https://www.worldclim.org/), including 19 climate variables (Bio1–Bio19). Future climate data include two climate models (the Coupled Model Intercomparison Project, Phase 6 (CMIP 6) and Global Climate Model of BCC-CSM2-MR (resolving power: 2.5 arc min [33,34,35,36,37])), two climate scenarios (SSPs: Shared Socioeconomic Pathways. SSPs126 corresponds to SSP1, which represents a sustainable development pathway, and RCP2.6, indicating that greenhouse gas emissions are limited to lower levels. Conversely, SSPs585 corresponds to SSP5, representing a fossil fuel-driven development pathway, and RCP8.5, which signifies a continuous increase in global greenhouse gas emissions), and four future time periods (2030s = 2020–2040; 2050s = 2040–2060; 2070s = 2060–2080; 2090s = 2080–2100).

Many variables exhibit spatial collinearity, which may lead to overfitting of the model that ultimately affects the prediction results. Therefore, we conducted a selection method for key environmental factors. Firstly, we performed pre-modeling experiments to input species distribution point data, current climate data, and future climate data (including 19 environmental variables) into the maximum entropy model software package (MaxEnt ver. 3.4.4). All parameters were kept constant by default, and the significance and contribution values of 19 climate factors were obtained (mean AUC by 10 model replicates = 0.894, TSS = 0.592) (Figure S1a). Secondly, the current data values of the species distribution points were extracted using ArcGIS ver. 10.8.1 and Pearson correlation for these 19 environmental variables by using the “reshape2” package in R ver. 4.2.1. Based on the importance and contribution values of the environmental variables in the pre-modelling, we standardized the Pearson values using the “pheatmap” package and used Euclidean distance as a distance measure to cluster 19 environmental variables into 6 categories, selecting one variable from each category (Bio3, Bio5, Bio6, Bio15, Bio18, Bio19) (Figure S1b). Next, we performed a Pearson correlation analysis (|r| < 0.8) again on the 6 categories of variables using the “reshape2” and “corrplot” packages (Figure S1c). Finally, we kept all parameters unchanged by default and entered these six sets of variables into the pre-modelling operation (ten repeated mean AUC = 0.897, TSS = 0.658), and the results showed that the accuracy of the model improved after the variable reduction.

### 2.4. Model Evaluation and Validation

We imported species distribution point data and environmental data into MaxEnt software for modeling operations. In this study, we used a total of 132 mountain apricot distribution points and 6 environmental variables, including 9 periods. In the modeling, we selected a random number of seeds, took 25% of the distribution points as the test set and the remaining 75% of the distribution points as the training set, and used the non-repeated sampling method for 10 repeated operations; the output value was in ASCII format. Additionally, the maximum number of background points was set to 10,000, and all other parameters were left at their default values. We analyzed the relative importance of each environmental variable, which includes the importance and contribution values of the environmental variables. In the test data, we used AUC-ROC values and the True Skill Statistic (TSS) to assess the credibility of the model. The range of AUC values was between 0.5 and 1. When the AUC is less than 0.6, the model is not credible; when the AUC is between 0.6 and 0.7, the model credibility is low; when the AUC is between 0.7 and 0.8, the model credibility is general; when the AUC is between 0.8 and 0.9, the model credibility is high; and when the AUC is between 0.9 and 1, the model credibility is very good. The TSS value ranges from −1 to 1, with values closer to 1 indicating better predictive performance. A value between 0.6 and 1 indicates good predictive performance [38,39,40].

### 2.5. Model Optimization

We used the Kuenm (https://github.com/marlonecobos/kuenm, last accessed on 27 March 2024) package to optimize the regularization multiplier and feature class parameters in R ver. 3.6.3 (https://www.r-project.org/) software [20]. We used 75% of the data as the training set in the modeling. A total of 1160 candidate models were evaluated with parameters reflecting all combinations of 40 regularization multiplier settings (from 0.1 to 4, an interval of 0.1) and 29 feature class parameters. Model selection was based on the order of statistical significance (partial ROC), predictive power (low omission rate), and complexity (AICc value). Firstly, the candidate models were filtered to retain those that were statistically significant; secondly, the model with a delta AICc value (<2) with significant and low omissions was selected; then, the model set was reduced using the omission rate criterion (i.e., <5%); finally, the eligible models were repeated 10 times to select the model with the largest average AUC value and TSS value.

### 2.6. Data Processing

We used the ArcGIS 10.8.1 software to classify and visualize the suitability of mountain apricot. The suitability threshold of the distribution area was predicted based on the MaxEnt model. The natural breakpoint method was used to classify the habitat suitability index of mountain apricot. The lowest level of threshold establishment was 0.086, and the distribution below this level was excluded [41]. The suitability grade of the mountain apricot distribution area was divided into unsuitable area (0–0.086), less suitable area (0.086–0.264), moderately suitable area (0.264–0.486) and highly suitable area (0.486–1). We fit the species distribution model based on current time, and then projected the fitted models into future climate scenarios. According to the classification of suitable areas, we calculated the area of suitable distribution in the current and future climate models (2030s = 2020–2040; 2050s = 2040–2060; 2070s = 2060–2080; 2090s = 2080–2100) in the “ggplot2” package in R ver. 4.2.1.

## 3. Results

### 3.1. Subsection

In total, we evaluated 1160 candidate models, with partial ROC < 0.05 and delta AICc < 2 for 4 models. We then incorporated the parameters from these four models into the MaxEnt analysis, yielding four sets of mean AUC and TSS: (0.884, 0.638), (0.896, 0.625), (0.882, 0.645), and (0.899, 0.670) (Table 1). Finally, the FC and RM parameter models with the largest mean AUC value were selected as the final model (FC = LQ, RM = 0.3, mean AUC = 0.899, TSS = 0.670), and the results showed that the model had good credibility.

### 3.2. Geographic Distribution of Mountain Apricots in China

**Figure 2:**
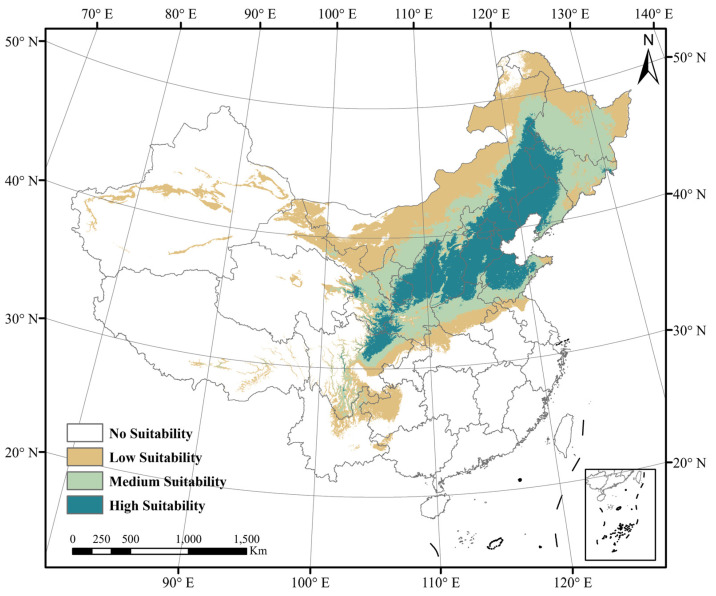
Suitable distribution area of *Prunus sibirica* by MaxEnt in China.

Under the current climate model, the total area of the mountain apricot’s suitable distribution area is 3,732,649 km^2^, of which the highly suitable distribution area is 958,073 km^2^, the moderately suitable distribution area is 1,148,046 km^2^, and the less suitable distribution area is 1,626,530 km^2^. The mountain apricot suitable area is distributed in 27 Chinese provinces and cities, mainly concentrated in northeast and northern China, with the highly suitable area concentrated in Beijing, Tianjin, Hebei, Shandong, Shanxi, Shaanxi, Liaoning, Western Jilin, Henan, Southern, Southern Gansu, Ningxia, Northern Sichuan, Heilongjiang and Eastern Qinghai (Figure 2).

### 3.3. Changes in Suitable Distribution Area Under Future Climate Change

**Figure 3:**
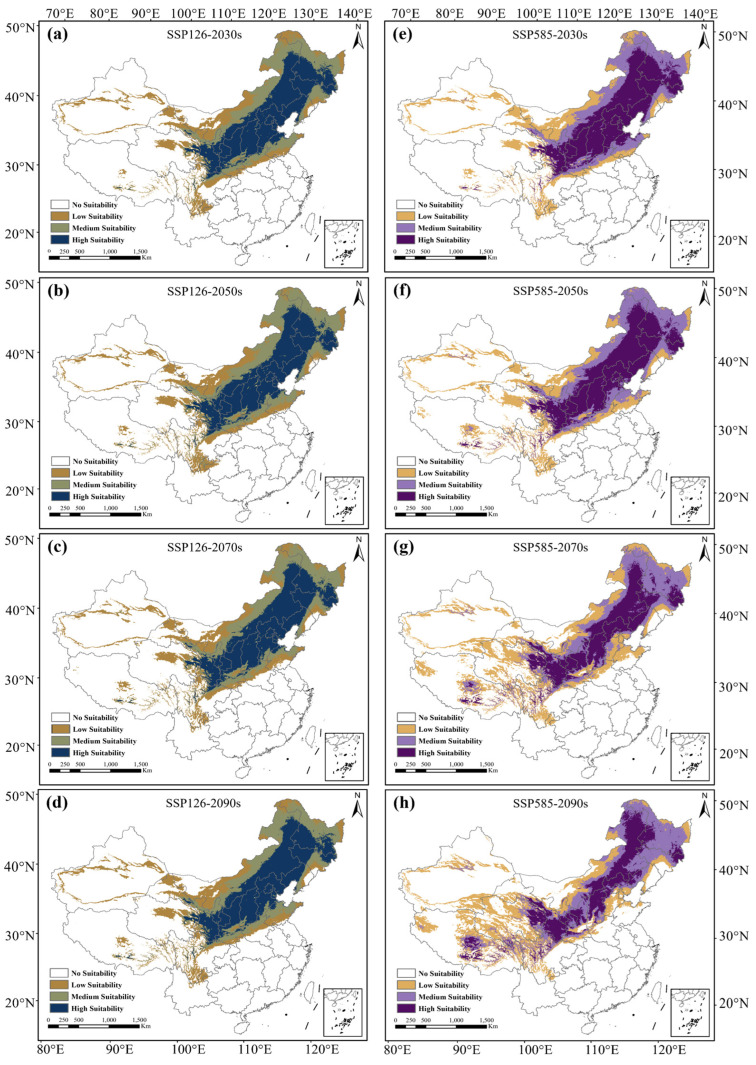
Suitable distribution area for *Prunus sibirica* in China in the future under different climate change scenarios. (**a**) Suitable distribution area for *Prunus sibirica* in China under SSP126−2030s scenario; (**b**) SSP126−2050s; (**c**) SSP126−2070s; (**d**) SSP126−2090s; (**e**) SSP585−2030s; (**f**) SSP585−2050s; (**g**) SSP585−2070s; (**h**) SSP585−2090s.

Under the two socioeconomic sharing models (SSPs126 and SSPs585), the main suitable distribution area of mountain apricot will still be concentrated in northeast China and north China in the next four periods of the 2030s, 2050s, 2070s and 2090s. In the low emission mode (SSPs126), the future mountain apricot highly suitable distribution area will still be concentrated in the 14 provinces and cities of Beijing, Tianjin, Hebei, Shandong, Shanxi, Shaanxi, Liaoning, Western Jilin, Northern Henan, Southern Gansu, Southern Ningxia, Northern Sichuan, Southwest Heilongjiang and Eastern Qinghai, and the current climate pattern for the mountain apricot’s highly suitable distribution area in provinces and cities will not change (Figure 3a–d). However, in the high emission mode (SSPs585), the change in the highly suitable distribution area will gradually shrink along the southeast and northwest direction, and extend in the northeast and southwest direction. After the 2090s, the highly suitable distribution area for mountain apricot will completely disappear in Shandong, and some highly suitable distribution areas for mountain apricot will appear in Tibet (Figure 3e–h).

**Figure 4:**
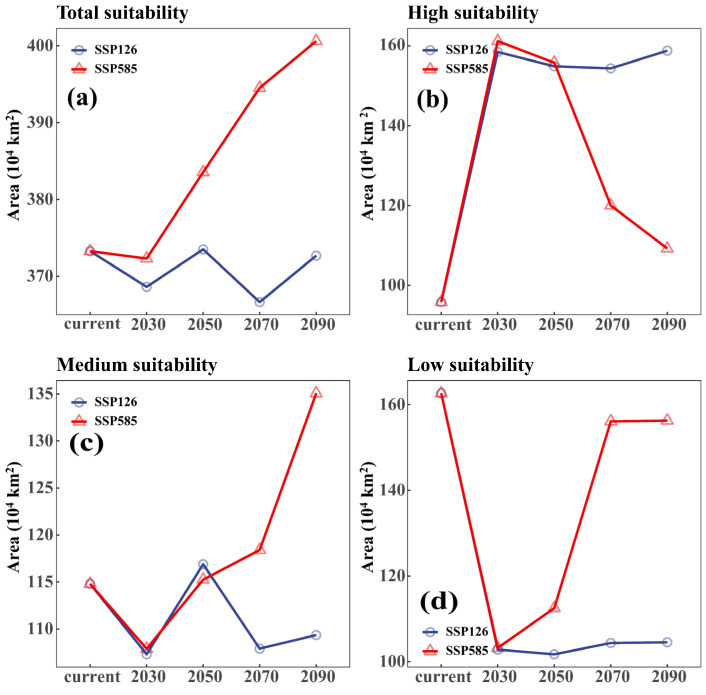
Changes in potentially suitable habitats from current to 2090 under two distinct climate change scenarios. (**a**) Total suitability; (**b**) High suitability; (**c**) Medium suitability; (**d**) Low suitability.

We also compared the area changes in the suitable distribution area between the two climate models. In the low emission mode of SSPs126, the change in the area of the suitable distribution was relatively stable. After the 2090s, the total suitable area would decrease by 5860 km^2^ (0.16%), but in the high emission mode of SSPs585, the total area of the suitable distribution area shows an increasing trend year by year. After 2090s, the total suitable area would increase by 273,459 km^2^ (7.33%) (Figure 4a). Moreover, area fluctuations in high-, moderate- and low-suitability areas are mainly concentrated in current–30s in the low emission mode (SSPs126), and maintain relative stability at 30s–90s. And the change values were 65.76%, −4.75% and −35.74%, respectively, by the 2090s and later. Area fluctuations in the high-, moderate- and low-suitability regions are similarly concentrated in current–30s in the high emission mode (SSPs585), but the high- and low-suitability regions would gradually tend towards current levels after significant changes in the 30s. By the 2090s and later, change values would be 14.02% and −3.91%, respectively. The moderate area would increase after the 30s, and would increase by 17.65% by the 2090s and later (Figure 4b–d).

### 3.4. Key Climate Factors

According to the jackknife test, the six environmental variables were ranked in order: Bio15 (precipitation seasonality: 34.9%), Bio3 (isothermality: 17.7%), Bio6 (min. temperature of coldest month: 15.0%), Bio19 (precipitation of coldest quarter: 12.1%), Bio18 (precipitation of warmest quarter: 11.5%), and Bio5 (max. temperature of warmest month: 8.7%) (Table S1).

The jackknife test results showed that each variable contributed to the model gain and was well validated in test gain and AUC (Figure S2). When the variables were used separately, the environmental variables with the highest gain were Bio5 (max. temperature of warmest month) and Bio18 (precipitation of warmest quarter). Specifically, omitting variables such as Bio5 or Bio18 resulted in a substantial reduction in the model’s gain, highlighting that these variables possess unique and indispensable information that is critical to the model’s overall predictive accuracy (Figure S2).

The best environmental conditions for the probability of occurrence in the study area were Bio3 = 27.02, Bio5 = 27.48 (°C), Bio6 = −11.93 (°C), Bio15 = 150.4 (mm), Bio18 = 342.82 (mm) and Bio19 = 0.09 (mm). The best environmental distribution range for mountain apricot occurrence probability was as follows: Bio3 = 23.04–30.94, Bio5 = 24.86–30.04 (°C), Bio6 = −20.21–−3.59 (°C), Bio15 = 106.06–150.40 (mm), Bio18 = 235.15–456.43 (mm) and Bio19 = 0–18.24 (mm) (Figure S3). Environmental distribution conditions were in the range Bio3 = 13.67–38.57, Bio5 = 20.82–33.99 (°C), Bio6 = −31.92–7.80 (°C), Bio15 = 55.46–150.40 (mm), Bio18 = 70.23–618.35 (mm) and Bio19 = 0–112.86 (mm) (Figure S3).

## 4. Discussion

As a significant fruit tree species in northern China, *Prunus sibirica* plays an important ecological role in water conservation, windbreak and sand fixation, desertification control and in maintaining the stability and multifunctionality of ecosystems [42]. Furthermore, the wild apricot’s suitable growth areas largely overlap with poverty-stricken mountainous regions in northern China, giving it substantial economic and social value in ecological afforestation efforts and poverty alleviation. However, due to climate change and overexploitation, wild apricot resources are facing the risk of significant depletion [21]. This study provides critical insights into the sustainable use and cultivation of wild apricot resources under future climate change scenarios.

### 4.1. Key Environmental Factors Influencing Wild Apricot Growth

Plant growth is influenced by multiple environmental factors, including temperature, precipitation, topography, and soil conditions. However, temperature and precipitation are generally considered the primary factors affecting plant growth [43,44]. Our study confirms that precipitation factors (Bio15, Bio19 and Bio18) and temperature factors (Bio3, Bio6 and Bio5) are key climatic variables influencing the growth and distribution of wild apricots, consistent with previous research findings [23,45].

Since temperature and precipitation are vital for photosynthesis, water metabolism and nutrient transport in plants, species richness tends to be lower in arid and semi-arid regions, with only drought- and cold-tolerant species emerging as dominant [46,47]. Our results also show that Bio15 (precipitation seasonality) and Bio3 (isothermality) are the most critical climatic factors influencing wild apricot growth and distribution. These two variables reflect the variability in precipitation and temperature, respectively, indicating that wild apricots possess strong adaptability to changes in both, which allows them to thrive in the harsh environments of northern China’s impoverished mountainous regions and become a dominant species in competitive ecosystems [48,49,50,51].

Comparing our results with other species, similar findings have been reported for several drought-tolerant species. For example, the distribution of *Pinus sylvestris* (Scots pine) in Europe is also heavily influenced by precipitation seasonality and temperature variability, with drought-tolerant genotypes thriving in regions with extreme climatic variability [52,53]. Similarly, *Quercus ilex* (holm oak) in the Mediterranean basin exhibits strong adaptability to fluctuating precipitation and temperature, demonstrating the ecological advantages of species that can adjust to both drought and cold conditions [54,55]. These comparisons highlight that wild apricot shares ecological strategies with other drought- and temperature-tolerant species, enabling its dominance in semi-arid ecosystems.

Previous physiological and molecular studies have highlighted the advantages of wild apricots, including their drought, cold and poor soil tolerance, rapid growth, extensive root systems and resistance to pests and diseases, making them a widely planted species. These traits also suggest that wild apricots are a primary candidate for reforestation and ecological restoration projects in the semi-arid loess hilly areas of China [56]. Our findings, from the perspective of ecological species distribution and interspecies competition, further corroborate these advantages.

### 4.2. Changes in Wild Apricot Distribution Under Future Climate Change

Using the MaxEnt model, this study simulated and predicted the potential distribution and range of wild apricots in China under future climate change scenarios (SSPs126 and SSPs585) from 2030 to 2100. The results indicate that, under the low-emission scenario (SSPs126), the total area of suitable wild apricot habitat remains relatively stable, with only a 0.16% reduction in the 2090s. This stability may be due to the minimal variability in precipitation and temperature under the low-emission scenario, maintaining relatively stable conditions for wild apricot growth. Similar results have been reported in previous studies; for example, under the SSPs126 scenario, the distribution of the endangered tree species *Keteleeria davidiana* also showed minimal changes due to the low variability in its key climate factors, temperature and precipitation [34]. Additionally, *Abies alba* (European silver fir) is another example of a species whose distribution is relatively stable under low-emission scenarios, as it also relies on consistent climatic conditions for regeneration and growth [57]. 

In contrast, under the high-emission scenario (SSPs585), the total suitable habitat for wild apricots showed a significant expansion, with a 7.33% increase by the 2090s. This expansion may be attributed to the species’ high ecological niche under extreme climate change, allowing wild apricots to outcompete other species and become dominant [48,58,59,60]. Specifically, under the high-emission scenario, the highly suitable habitats for wild apricots would gradually shrink in the southeast and northwest, while expanding towards the northeast and southwest. This geographical shift is likely related to changes in temperature and precipitation patterns due to global warming [2,42]. The existence of mountainous terrain in these directions, compared to the plains, likely plays a role in amplifying climate variability, allowing wild apricots, with their strong resilience, to succeed in these competitive environments [21,43]. This shift is similar to patterns observed in other species. For example, *Pinus nigra* (black pine) has been found to expand its range in higher altitudes under similar high-emission scenarios, as it can better cope with the increasing temperature and changing precipitation patterns [61]. In the case of *Fagus sylvatica* (European beech), an important broadleaf species, studies have predicted a substantial range contraction in southern Europe and an expansion into more northern regions under future climate change, particularly under high-emission scenarios. This pattern mirrors our findings for wild apricot, where regions currently considered highly suitable may shift geographically, with changes in the species’ potential distribution driven by the intensity of climate change [62].

Our findings have important implications for wild apricot cultivation and management, especially in providing a scientific basis for its sustainable use to increase farmers’ income in northern poverty-stricken regions under future climate change. Additionally, with the ongoing shifts in global climate, the distribution of wild apricots is expected to undergo significant migrations. For instance, under the high-emission scenario, highly suitable habitats for wild apricots are projected to disappear entirely in Shandong Province, while new suitable areas may emerge in Tibet. Our study offers valuable guidance for local governments in addressing ecosystem species loss and in introducing ecologically and economically beneficial tree species under climate change.

## 5. Conclusions

In conclusion, this study successfully predicted the current and future potential distribution of wild apricots using an optimized MaxEnt model. The results indicate that climate change will significantly impact the distribution of wild apricots, particularly under high-emission scenarios, where their suitable habitats are expected to shift considerably. These findings provide valuable insights into the conservation and management of wild apricots, while also contributing to a broader understanding of how climate change influences plant distributions.

Future research should explore the effects of other environmental factors and the interactions between plants and animals [48] and human activities on wild apricot distribution to comprehensively assess habitat suitability dynamics. Furthermore, integrating more climate models and scenarios can improve the accuracy and reliability of predictions, offering precise data support for ecosystem management and biodiversity conservation [63].

## Figures and Tables

**Table 1:** Evaluation results of the MaxEnt model under different parameter settings.

Regularization Multiplier	Feature Combination	Omission Rate at 5%	Delta AICc	Mean AUC	TSS
0.4	LQP	0.061	0	0.884	0.638
0.1	LQ	0.061	0.683	0.896	0.625
0.2	LQ	0.061	1.155	0.882	0.645
0.3	LQ	0.061	1.669	0.899	0.670

## Data Availability

Dataset available upon request from the authors.

## Supplementary Materials

The following supporting information can be downloaded at: https://www.mdpi.com/article/10.3390/biology13120973/s1, Figure S1: Selection method for key environment variables. (a) Contribution and significance values of each environmental variable in the pre-modeling, where the red font is the final selected key environmental variable. (b) Results of cluster analysis of Pearson correlation coefficients for 19 environmental variables after standardization. (c) Pearson correlation coefficients for the 6 key environmental variables in the final model. Table S1: Contribution (%) of the leading five environmental variables in species distribution modeling of *Prunus sibirica* in the study area. Figure S2: Importance of environment variables to *Prunus sibirica* using jackknife analysis. Figure S3: MaxEnt model response curves of the six bioclimatic variables used in the predictive species distribution modeling of *Prunus sibirica* in China.
