# Supplementary material for: Prediction of the Future Evolution Trends of Prunus sibirica in China Based on the Key Climate Factors Using MaxEnt Modeling
Source: Biology (Basel). 2024 Nov 25;13(12):973. doi: 10.3390/biology13120973 (PMC11672940; doi:10.3390/biology13120973)
Supplement: Supplementary file 1 [file biology-13-00973-s001.zip › biology-3297697-supplementary.pdf]

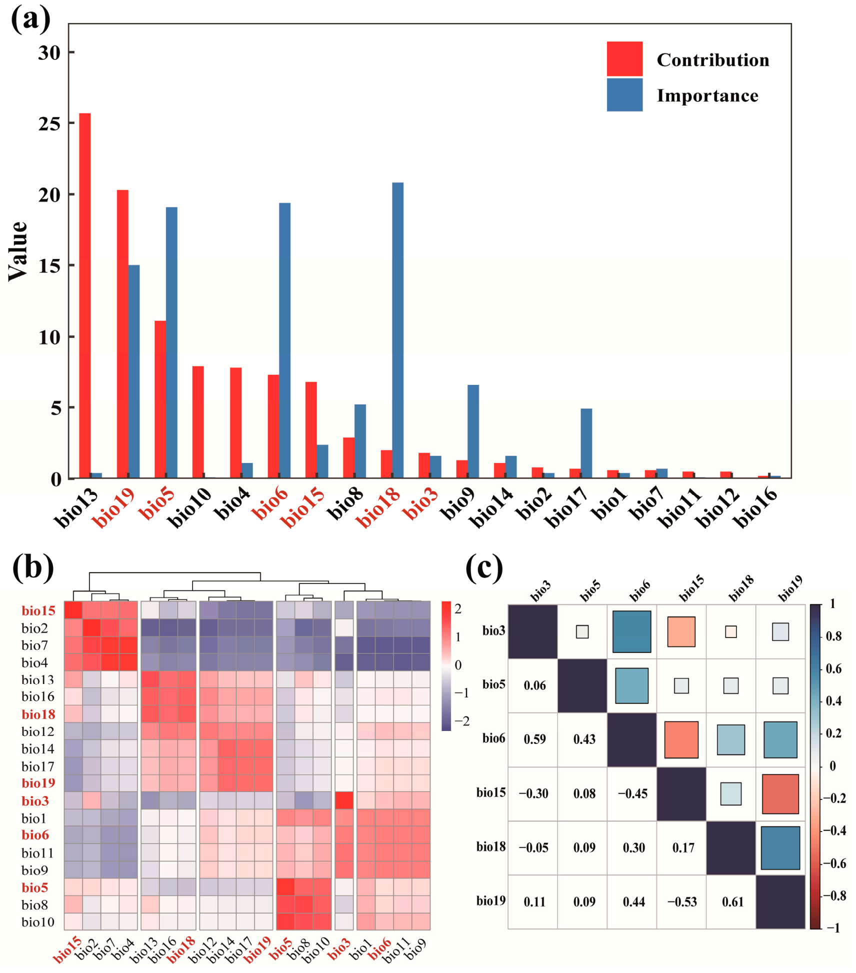

Figure S1. Selection method for key environment variables. (a) Contribution and significance values of each environmental variable in the pre-modeling, where the red font is the final selected key environmental variable. (b) Results of cluster analysis of Pearson correlation coefficients for 19 environmental variables after standardization. (c) Pearson correlation coefficients for the 6 key environmental variables in the final model.

Table S1. Contribution (%) of the leading five environmental variables in species distribution modeling of *Prunus sibirica* in the study area.

Variable	Code	Percentage contribution
Precipitation seasonality (coefficient of variation) (mm)	Bio15	34.9
Isothermality (BIO2/BIO7) (×100)	Bio3	17.7
Min. temperature of coldest month (° C)	Bio6	15.0
Precipitation of coldest quarter (mm)	Bio19	12.1
Precipitation of warmest quarter (mm)	Bio18	11.5
Max. temperature of warmest month (° C)	Bio5	8.7

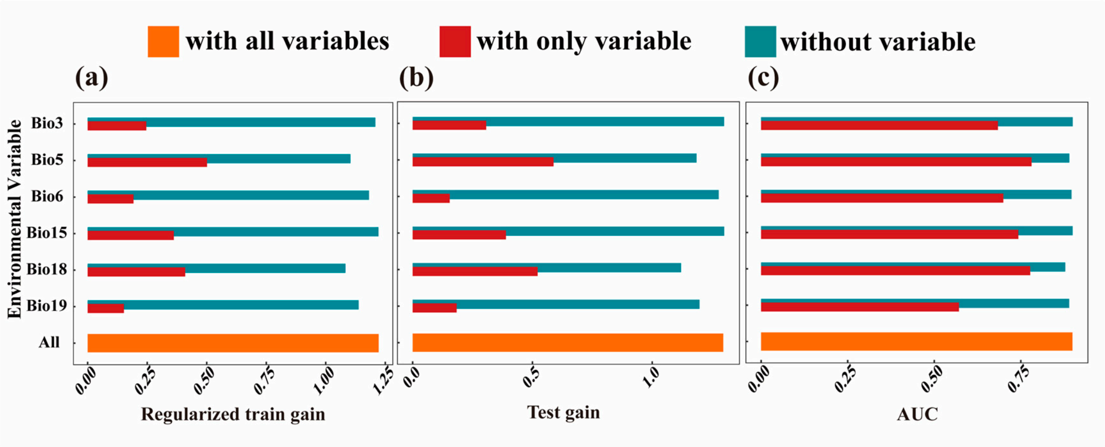

Figure S2. Importance of environment variables to *Prunus sibirica* using jackknife analysis.

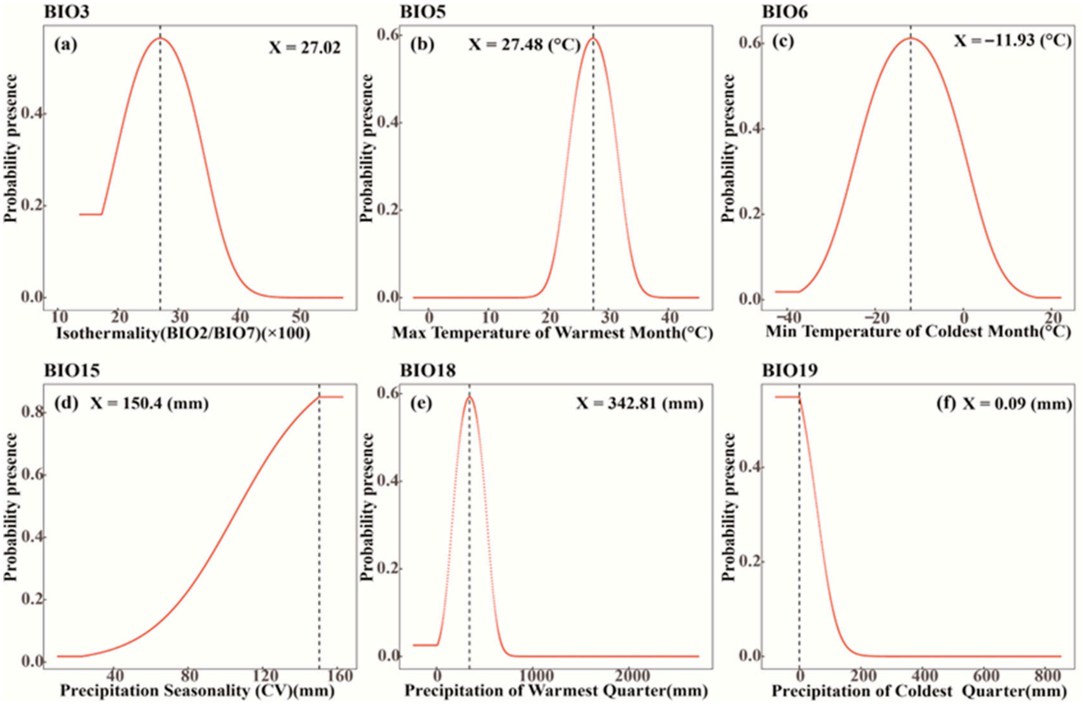

FigureS3. MaxEnt model response curves of the six bioclimatic variables used in the predictive species distribution modeling of *Prunus sibirica* in China.
